# Biological effects of human placental extracts - variations in manufacturing methods and compositions

**DOI:** 10.3389/fphar.2025.1707890

**Published:** 2025-12-10

**Authors:** Yikelamu Alimu, Takeshi Yamamoto, Yasukazu Nakahata

**Affiliations:** 1 Corporate Planning Department, Melsmon Pharmaceutical Co. Ltd., Tokyo, Japan; 2 Department of Integrative Neuroscience, Graduate School of Biomedical Sciences, Nagasaki University, Nagasaki, Japan

**Keywords:** Melsmon, Laennec, Placentrex, human placental extracts, manufacturing methods, biological effects

## Abstract

Placental extracts have been used in traditional medicine across many cultures for centuries. In Traditional Chinese Medicine and Japanese Kampo, placenta has been valued for its ability to reduce fatigue, promote recovery, and enhance vitality. This stems from the belief that the placenta possesses regenerative and life-sustaining properties. In the modern era, the therapeutic use of placenta began with Filatov’s “tissue therapy” in the Soviet Union, leading to the development of various extraction techniques from both human and animal placentas. Today, a variety of placental extracts has been manufactured using placentas from humans and livestock. The placental extracts that are currently being manufactured have different compositions owing to differences in origin, placental part used, and manufacturing methods. Despite the differences in the contents of these placental extracts, it has been shown that they exert, to some degree, common biological activities, such as antioxidant, anti-inflammatory, and immunomodulatory functions. Therefore, placental extracts are used as pharmaceuticals to treat menopausal disorders, improve liver function, treat osteoarthritis, and promote wound healing. They are also used as dietary supplements to improve skin texture and for anti-aging purposes. Notably, the relationship between the biological effects of a placental extract and its contents has not been accurately and comprehensively understood. This review summarizes the biological effects and functions of various placental extracts that have been reported to date, including our recent findings, and provides an overview of the biological effects of some human placental extracts for which we were able to obtain the manufacturing method information.

## 1 Introduction

Many placental extracts have been manufactured using human and livestock placentas as starting materials and utilized as pharmaceuticals and supplements for a long time (Pan et al., 2017; Shen et al., 2022b). Placental extracts were initially used for “tissue therapy” by Filatov in the Soviet Union (Gromova et al., 2022). Based on Filatov’s studies on “organotherapy,” many new studies have been conducted, and extraction methods have been developed to manufacture extracts from various animal placentas, including human placentas. Placental extracts have been mainly utilized in Asia as pharmaceuticals and beauty supplements (Pan et al., 2017; Shen et al., 2022b; Gwam et al., 2023). To date, more than 5,000 basic and clinical studies have reported on placental extracts (Gromova et al., 2022), and shown that human placental extracts exhibit various effects on the body (Pan et al., 2017; Gromova et al., 2022; Shen et al., 2022b; Gwam et al., 2023). In addition, placental extracts from animals other than humans, specifically those from pigs, horses, and sheep, also exhibit various positive effects on the body.

Table 1 shows the origins of the placentas used to prepare the major placental extracts as well as the relationship between the effects and functions of the placental extracts from various animals. Placental extracts have been reported to have various effects, including antioxidant (Togashi et al., 2000; Watanabe et al., 2002; Choi et al., 2014a; Park et al., 2015; Tang et al., 2015; Bak et al., 2019; Ghoneum and El-Gerbed, 2021; Laosam et al., 2021; Huang et al., 2022), anti-inflammatory (Kim et al., 2015; Akagi et al., 2016; Heo et al., 2018; Tebakari et al., 2018; Yoon et al., 2020; Flannery et al., 2021; Ghoneum and El-Gerbed, 2021; Xu et al., 2023), cell proliferation/differentiation (Wo et al., 2023; Wu et al., 2023; Ye et al., 2024), antimicrobial (Goswami et al., 2017; Laosam et al., 2021), and neurogenesis effects (de Toledo et al., 2021; Ye et al., 2024). Furthermore, many *in vitro*, *in vivo*, and clinical studies have shown that placental extracts improve liver function (Choi et al., 2014b; Shimokobe et al., 2015; Liu et al., 2018; Kim et al., 2020a; Ghoneum and El-Gerbed, 2021; Kim et al., 2022; Shen et al., 2024), fatigue (Lee et al., 2012; Park et al., 2016; Yoon et al., 2020), skin conditions (Hong et al., 2015; Park et al., 2017; Aioi et al., 2021; Huang et al., 2022; Nagae et al., 2022; Shen et al., 2022a), knee osteoarthritis (Park and Cho, 2017; Flannery et al., 2021), wound healing (Hong et al., 2010; Goswami et al., 2017; Singh and Bhattacharyya, 2017), and menopausal disorders (Karasawa et al., 1981a; Kim et al., 2013; Choi et al., 2022; Kim et al., 2023). Clinical studies have shown that placental extracts exert additional positive effects on humans, such as hair growth promotion (Kang et al., 2014; Kwon et al., 2015; Kim et al., 2020b), improvements in depression (Kovalenko and Atalyan, 2016; Ye et al., 2024), milk secretion deficiency (Karasawa et al., 1981b), and memory impairment (de Toledo et al., 2021).

**TABLE 1:** Effects and functions of placental extracts from different origins.

Placental origin Placental extract functions/effects	Human	Porcine	Equine	Caprine	Bovine
Antioxidative	Huang et al. (2022); Togashi et al. (2000); Watanabe et al. (2002); Bak et al. (2019); Ghoneum and El-Gerbed (2021)	Choi et al. (2014a); Tang et al. (2015); Park et al. (2015); Laosam et al. (2021)			
Anti-inflammatory	Ghoneum and El-Gerbed (2021); Akagi et al. (2016); Flannery et al. (2021); Xu et al. (2023)	Kim et al. (2015); Tebakari et al. (2018); Heo et al. (2018); Yoon et al. (2020)			
Cell proliferation/Cell differentiation	Wo et al. (2023); Wu et al. (2023)	Ye et al. (2024)			
Antibacterial	Goswami et al. (2017)	Laosam et al. (2021)			
Neurogenesis		Ye et al. (2024)	de Toledo et al. (2021)		
Improvement of liver function	Ghoneum and El-Gerbed (2021); Shimokobe et al. (2015); Choi et al. (2014a)	Kim et al. (2020a); Kim et al. (2022)		Liu et al. (2018)	Shen et al. (2024)
Improvement of fatigue	Park et al. (2016); Lee et al. (2012)	Yoon et al. (2020)			
Improvement of skin condition	Huang et al. (2022)	Hong et al. (2015); Park et al. (2017); Aioi et al. (2021)	Nagae et al. (2022)		
Improvement of osteoarthritis	Flannery et al. (2021); Park et al. (2017)				
Wound healing	Ghoneum and El-Gerbed (2021); Hong et al. (2010); Singh and Bhattacharyya (2017)				
Improvement of menopausal symptoms	Kim et al. (2013); Kim et al. (2023); Choi et al. (2022); Karasawa et al. (1981a)				
Hair growth promotion	Kang et al. (2014); Kwon et al. (2015); Kim et al. (2020b)				
Improvement of depression	Kovalenko and Atalyan (2016)	Ye et al. (2024)			
Improvement of hypogalactia	Karasawa et al. (1981b)				
Prevention of memory disorders			de Toledo et al. (2021)		

The placenta is the organ formed in the uterus of female placental mammals (Eutheria) that connects the mother and fetus during gestation. In addition, the placenta functions as a temporary endocrine gland that secretes protein hormones, such as chorionic gonadotropin and placental lactogen, steroid hormones, such as estrogen and progesterone, and peptide hormones produced in the hypothalamus (Burton and Fowden, 2015; Khorami-Sarvestani et al., 2024). It also produces growth factors related to wound healing (Khorami-Sarvestani et al., 2024). Therefore, placental extracts are composed of a large number of components, including the above-mentioned biological elements produced by the placenta as well as proteins, lipids, and carbohydrates that make up the placental tissue, the degradation products of these components, and the trace elements present in the tissue (Pan et al., 2017; Shen et al., 2022b; Gwam et al., 2023).

Resulting from the rich composition of the placenta, placental extracts have shown beneficial effects on the human body (Pan et al., 2017; Gromova et al., 2022; Shen et al., 2022b; Gwam et al., 2023). However, the contents of the placental extracts currently used as pharmaceuticals and supplements have not been fully elucidated, and only a small part of the relationship between the components and their biological and beneficial effects on the human body has been clarified (Pan et al., 2017; Shen et al., 2022b; Gwam et al., 2023). For example, a comparison between human placental extracts prepared by hydrolysis with hydrochloric acid and those hydrolyzed with proteases showed that the main components of the former are low molecular weight compounds, whereas those in the latter are high molecular weight compounds (Inoue et al., 2015). A comparison of the DNA synthesis-promoting effects of these two types of placental extracts revealed that the extract obtained by hydrolysis with hydrochloric acid promoted DNA synthesis more effectively than that obtained by hydrolysis with proteases (Inoue et al., 2015). Even if the placentas derive from the same source, the effects of the extracts on the body depend on the manufacturing method, suggesting that these differences are due to variations in the final composition of the extracts (Inoue et al., 2015).

For these reasons, research is being actively conducted to elucidate the active components and mechanisms underlying placental extract function in the body as well as to utilize the various effects of placental extracts in the treatment of diverse diseases and illnesses (Flannery et al., 2021; Torshin et al., 2023; Tebakari et al., 2024; Tian et al., 2024). Therefore, knowledge in this regard is being gradually accumulated (Torshin et al., 2023; Tebakari et al., 2024; Tian et al., 2024). However, this knowledge has not been comprehensively or systematically organized, and the findings are not widely known. Furthermore, the biological effects of placental extracts, which differ in their origin and manufacturing methods, are not fully understood.

In this review, we focus on placental extracts derived from the human placenta and outline the research on their preparation methods and contents as well as their biological and beneficial effects on the human body. For this review, relevant literature was collected through comprehensive searches of PubMed using the keyword “Human placental extracts” for publications published from 2014 to 2025. In addition, separate searches were conducted using the keywords “Melsmon”, “Laennec”, and “Placentrex” on Google Scholar to identify further relevant studies, respectively. Among the articles retrieved through these searches, those accessible to us were selected and included in the present review.

## 2 Manufacturing methods for human placental extracts

**FIGURE 1:**
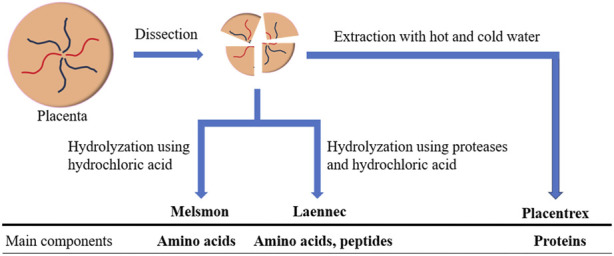
Schematic representation of the manufacturing methods of three human placental extracts: Melsmon, Laennec, and Placentrex. Extract manufacturing methods are broadly divided into two groups. Melsmon and Laennec production includes a hydrolysis process, whereas that of Placentrex does not (these manufacturing methods are described in Section 2).

Table 1 summarizes the relationship between various placental extracts and their effects and functions. However, these placental extracts are not manufactured using the same method. Thus, their contents are not identical. In this section, we outline those human placental extract manufacturing methods that can be confirmed in scientific reports and/or patent information. The human placental extracts used in this review are Melsmon (Melsmon Pharmaceutical Co., Ltd., Tokyo, Japan), Laennec (Japan Bio Products Co., Ltd., Tokyo, Japan), and Placentrex (Albert David Ltd., Kolkata, India), which have been broadly classified into two groups. One group includes Melsmon and Laennec, whose manufacturing process includes a hydrolysis step, and the other includes Placentrex, whose manufacturing process does not include hydrolysis of the placental tissues. A simple scheme for the manufacturing of these three human placental extracts is shown in Figure 1.

### 2.1 Manufacturing method of Melsmon

Melsmon has been shown to be effective in treating menopausal disorders and milk secretion deficiency (Karasawa et al., 1981a; Karasawa et al., 1981b), and the Japanese Ministry of Health, Labour and Welfare has approved the production of this pharmaceutical. The manufacturing method for Melsmon is summarized as follows: first, a frozen term placenta confirmed to be negative for viral and bacterial infections by serological testing is used. Further, the umbilical cord tissue is then removed, and the villous portion of the placenta is subjected to a de-blooding process, followed by extraction and purification through hydrochloric acid hydrolysis. The pH is then adjusted to 7.0 and benzyl alcohol is added. After the concentrations of the components are balanced, the extract is packed in ampoules and sterilized with high-pressure steam.

### 2.2 Manufacturing method of Laennec

Laennec is a medicine that was first approved for manufacture by the Japanese Ministry of Health, Labour and Welfare for treating “cirrhosis of the liver,” and whose efficacy was later reevaluated. Currently, it is also indicated for the “improvement of liver function in chronic liver disease.”

The following is a summary of the manufacturing method of Laennec, which involves mixing solutions obtained by hydrolyzing the human placenta with pepsin and hydrochloric acid (Kimoto et al., 2024): The placenta from a normal human delivery is refrigerated at 2 °C–4 °C for approximately 4 days, washed and dissected into small pieces, homogenized with the addition of acetone, defatted, and dried. An acidic solution (pH 2.0) using the dried placenta fragments is then prepared by adding hydrochloric acid. This solution is then digested with pepsin for a day and a night and collected by centrifugation. The precipitate obtained via centrifugation is then heated with hydrochloric acid for hydrolysis. This hydrolyzed solution is filtered through activated charcoal and mixed with the liquid fraction of the pepsin-digested solution (*i.e.*, the supernatant obtained by centrifugation), and the hydrochloric acid is removed using an anion exchange resin. Finally, the pH is adjusted to 6.1–6.4 using a NaOH solution (Kimoto et al., 2024).

### 2.3 Manufacturing method of Placentrex

The method for manufacturing Placentrex was reported by Chakraborty and Bhattacharyya (2012). After removing the umbilical cord and amniotic membrane from a fresh placenta that has been tested for HIV antibodies and hepatitis B surface antigen to confirm its safety, the placenta is dissected into small pieces. These fragments are then divided into two groups. One of the groups is incubated at 90 °C and the other at 6 °C, before undergoing a single hot- and cold-water extraction. The resulting extracts are then mixed, sterilized with saturated steam for 40 min, and combined with 1.5% (v/v) benzyl alcohol (as preservative); the extract is packed in ampoules and sterilized once again with saturated steam for 20 min (Chakraborty and Bhattacharyya, 2012).

## 3 Composition of human placental extracts

### 3.1 Melsmon composition

Park et al. (2010) reported that Melsmon is mainly composed by the following 16 amino acids: 0.965 mg of glutamic acid, 0.495 mg of aspartic acid, 0.483 mg of leucine, 0.468 mg of glycine, 0.370 mg of arginine, 0.362 mg of alanine, 0.312 mg of lysine, 0.283 mg of serine, 0.275 mg of proline, 0.232 mg of threonine, 0.205 mg of valine, 0.142 mg of phenylalanine, 0.133 mg of tyrosine, 0.130 mg of isoleucine, 0.057 mg of histidine, and 0.055 mg of methionine in 1 mL. Besides, 100 g of Melsmon contain 0.23 g of *N*-acetylneuraminic acid, 0.02 g of *N*-glycolylneuraminic acid, 0.10 g of glucosamine, 0.05 g of galactosamine, and 0.01 g of glucose, and stearic acid (18:0) and oleic acid [18:1(n-9)] as major saturated and unsaturated fatty acids (of the total extractable lipids), respectively (Park et al., 2010). Besides, the Melsmon Pharmaceutical website indicates that one ampoule (2 mL) of Melsmon contains 100 mg of water-soluble components and 0.03 mL of benzyl alcohol.

### 3.2 Laennec composition

The components of Laennec, reported by Nakayama et al. (Nakayama et al., 1989a), a total of 18 amino acids were identified, including the following 11 amino acids: 7.96 mg of glutamic acid, 6.44 mg of aspartic acid, 6.38 mg of glycine, 3.42 mg of alanine, 3.06 mg of leucine, 2.1 mg of serine, 1.58 mg of threonine, 1.18 mg of methionine, 1.0 mg of valine, 0.86 mg of phenylalanine, and 0.82 mg of isoleucine in 1 mL (Nakayama et al., 1989a). Laennec also contains pyroglutamic acid, which is formed by the intramolecular condensation of the carboxyl and amino groups of glutamic acid, forming a lactam ring (Inoue et al., 2015). In addition, many studies have searched for the biologically active compounds in Laennec. The results show that Laennec contains dipeptides, such as *cyclo-trans*-4-L-hydroxyprolyl-*L*-serine (Wu et al., 2008a) and *trans*-4-*L*-hydroxyprolyl-*L*-serine (Liu et al., 2000), that exert strong antihepatitis activities (Liu et al., 2000). Furthermore, Torshin et al. conducted 20 proteomics experiments using Laennec and identified 41 peptides consisting of 4–8 amino acid residues (Torshin et al., 2023); they suggested that the enzymatic activities of caspases, such as caspase-1, caspase-3, and caspase-4, which are essential enzymes in the apoptosis pathway, and mitogen-activated protein kinases (MAPK), which function as key molecules that transmit extracellular signals to the nucleus, might be regulated by such peptides (Torshin et al., 2023). Besides, the website of Japan Bio Products Co., Ltd. indicates that one ampoule (2 mL) of Laennec contains 112 mg of water-soluble substances. Unlike Melsmon, Laennec does not contain benzyl alcohol.

### 3.3 Placentrex composition

As described in Section 2.3, the manufacturing process of Placentrex does not involve hydrolysis of placental tissues with hydrochloric acid or proteases. Therefore, the main components of Placentrex differ from those of Melsmon and Laennec; they are mainly proteins. Sur et al. reported that Placentrex contains proteins (0.95 g/L), DNA (2.8 mg/L), RNA (1.6 mg/L), Na^+^ ions (27.9 mmol/L), K^+^ ions (3.07 mmol/L), and Cl^−^ ions (15.1 mmol/L) (Sur et al., 2003). However, the identity and functions of the proteins contained in Placentrex remain unknown.

## 4 Biological functions of human placental extracts

We next discuss the effects and functions of Melsmon, Laennec, and Placentrex, which are summarized in Tables 2–4, respectively.

**TABLE 2:** Biological functions and effects of Melsmon.

Study type	Effects	Ref.
*In vitro*	Increased production of collagen type 1 in primary human gingival fibroblasts Inhibition of IL-6 and IL-8 secretion from primary human gingival fibroblasts	Akagi et al. (2016)
	Significantly enhanced cell proliferation, alkaline phosphatase activity, and odontogenic differentiation Combination with MTA promoted angiogenic activity through the activation of key signaling pathways, including Akt, mTOR, and MAPK	Chang et al. (2016)
	Increased the expression of proliferation and regeneration-related markers such as Ki-67, cathelicidin, and sirtuins 1 and 6	Kvetnoy et al. (2019)
	Increased expression of extracellular matrix/structural construction-related genes, such as *COL1A1, COL5A3, ELN,* and *HAS2* in NHDF cells	Chang et al. (2022)
	Increased expression of the antioxidant genes *CYGB, APOE, NQO1*, and *PTGS1* Increased expression of NRF2 and oxidative stress alleviation through the activation of the NRF2-mediated pathway Improvements in skin aging-associated damage	Huang et al. (2022)
*In vivo*	Wound healing promotion after direct administration to the wound edge	Hong et al. (2010)
	Reduction in DNA damage induce by exposure to benzo[a]pyrene (BaP) Suppression of the oxidative damage and secretion of inflammatory cytokines caused by exposure to BaP	Park et al. (2010)
	Attenuation of DNA damage caused by acute gamma-ray exposure	Oh et al. (2023)
Clinical	Hypogalactia improvements	Karasawa et al. (1981b)
	Improvements in menopausal symptoms	Kim et al. (2013); Kim et al. (2023); Choi et al. (2022); Karasawa et al. (1981a); Orazov et al. (2017a)
	Decreased times to fall asleep and nighttime awakening numbers Extension of sleep duration	Kim et al. (2013); Kim et al. (2023); Kovalenko and Atalyan (2016)
	Improved average hair thickness and hair count	Kang et al. (2014)
	Improved mental and emotional symptoms such as depression and mood swings Improved skin conditions	Kovalenko and Atalyan (2016); Pokul et al. (2017)
	Improved urogenital syndrome	Orazov et al. (2017a)
	Improved regulation of the ovary menstrual cycle	Orazov et al. (2017b)

**TABLE 3:** Biological functions and effects of Laennec.

Study type	Effects	Ref.
*In vitro*	Inhibition of the leakage of hepatic cytosolic enzymes	Wu et al. (2008a); Liu et al. (2000); Wu et al. (2008b)
	Suppression of hepatocyte apoptosis and ICAM-1 expression	Wu et al. (2008a)
	Promotion of DNA synthesis in primary rat hepatocytes via MAPK signaling	Inoue et al. (2015)
	Inhibition of the proliferation of HepG2 in a dose-dependent manner via the Akt pathway	Yamaguchi et al. (2016)
	Increased antioxidant gene expression via NRF2 activation Improved hepatic fibrosis	Yamauchi et al. (2020); Shin et al. (2019)
	Wound healing by inducing *Col1, Scx*, and *Tnmd* gene expression	Shin et al. (2019)
	Promoted the polarization of macrophages toward the anti-inflammatory M2 phenotype and activated anti-inflammatory signaling pathways	Ishikawa et al. (2025)
*In vivo*	Promotion of liver regeneration. Improvement of liver injury Prevention of immune-mediated liver damage	Nakayama et al. (1989a); Nakayama et al. (1989b); Liu et al. (2000); Wu et al. (2008b); Yamauchi et al. (2020); Yang et al. (2009); Yamauchi et al. (2019a); Ishikawa et al. (2025)
	Tumor cell-death induction, tumor growth inhibition	Yamaguchi et al. (2016)
	Anti-stress effects via the regulation of nitric oxide (NO) synthase and antioxidant activity in the brain	Park et al. (2018)
	Suppression of weight loss due to SARS-CoV-2 infection and replication of the virus in the body	Kim et al. (2021)
	Inhibition of the expression of the heart failure-related genes BNP and βMHC and of the heart remodeling-related genes collagen α1, TGF-β, and MMP2 Suppression of inflammatory cell infiltration and the expression of the inflammatory-related molecules TNF-α and ICAM-1	Yamauchi et al. (2019b)
	Suppression of the immune response in damaged ligaments, promoting their healing	Shin et al. (2019)
Clinical	Reduction in chronic fatigue and improved physical endurance Improved physical pain, vitality, emotional state, mental health, and QOL	Glazachev et al. (2017)
	Significantly improves pain and shoulder function in impingement syndrome	Kim et al. (2025)

**TABLE 4:** Biological functions and effects of Placentrex

Study type	Effects	Ref.
*In vitro*/*In vivo*	Inhibition of platelet aggregation Suppression of acute and subacute inflammation	Sur et al. (2003)
Clinical	Slow down mucositis progression, reducing treatment interruptions and pain, and improving dysphagia	Kondaveeti et al. (2018)
	Reduction in the severity of radiation-induced mucositis Reduction in the number of interruptions or delays in head and neck malignant tumor treatment	Prasad et al. (2024)
	Suppression of the typical symptoms of oral submucosal fibrosis such as the sensation of burning	Shinde et al. (2019); Kisave et al. (2020); Reddy et al. (2022); Ek et al. (2023)

### 4.1 The functions/effects of Melsmon

#### 4.1.1 Functions/effects confirmed *in vitro*

Kvetnoy et al. investigated the molecular mechanisms underlying the anti-aging effects of the human placental hydrolysate preparation Melsmon in cultured human skin fibroblasts (Kvetnoy et al., 2019). Their results showed that Melsmon increased the expression of proliferation and regeneration-related markers such as Ki-67, calreticulin, and sirtuins 1 and 6. These findings indicate that Melsmon exerts strong geroprotective and regenerative properties by stimulating cellular activity and delaying senescence processes *in vitro*.

The research carried out by Akagi et al. in human primary gingival fibroblasts demonstrated that Melsmon increased the production of collagen type 1, which is related to the regenerative ability of periodontal tissue, and inhibited the secretion of the lipopolysaccharide (LPS)-induced inflammatory cytokines interleukin (IL)-6 and IL-8 (Akagi et al., 2016). Based on these results, Akagi et al. concluded that Melsmon can modulate the function of human gingival fibroblasts. In addition, we reported that Melsmon increased the expression of most of the genes related to extracellular matrix (ECM)/structural construction, such as *COL1A1*, *COL5A3*, *ELN*, and *HAS2*, in normal human dermal fibroblasts (NHDF) (Chang et al., 2022). Furthermore, Melsmon treatment increased the levels of collagen type 1, proteoglycan, elastin, and hyaluronic acid in these cells. According to these results, we concluded that Melsmon treatment activates the expression of ECM-related genes in NHDF cells.

We also studied the effects of Melsmon on cellular senescence in NHDF under oxidative stress conditions. We found that Melsmon enhanced the expression of the antioxidant genes *CYGB*, *APOE*, *NQO1*, and *PTGS1* (Huang et al., 2022). In addition, Melsmon treatment increased the protein levels of nuclear factor erythroid 2-related factor 2 (NRF2), an important molecule in the antioxidant pathway in NHDF under oxidative stress. Based on these results, we concluded that Melsmon treatment delays cellular senescence by alleviating oxidative stress through the upregulation of the NRF2-mediated antioxidant pathway, suggesting an improvement in the damage associated with skin aging.

Furthermore, Chang et al. examined the combined effects of mineral trioxide aggregate (MTA) and Melsmon on cultured human dental pulp cells and demonstrated that Melsmon significantly enhanced cell proliferation, alkaline phosphatase activity, and odontogenic differentiation (Chang et al., 2016). Moreover, the combination of Melsmon and MTA promoted angiogenic activity through the activation of key signaling pathways, including Akt, mTOR, and MAPK. These *in vitro* findings indicate that Melsmon can stimulate cellular proliferation and differentiation while enhancing angiogenesis, suggesting its potential role in tissue regeneration and repair.

#### 4.1.2 Functions/effects confirmed *in vivo*

Hong et al. studied the effects of Melsmon on wound healing and its biochemical mechanisms (Hong et al., 2010). Single 8-mm full-thickness skin defects were created on the backs of mice by punch biopsy, and wound healing was evaluated in Melsmon treated and saline treated control groups using digital images taken every 3 days for 2 weeks; a 0.2 mL injection around each wound was prepared by diluting one 2 mL ampoule of Melsmon with 200 mL of 0.9% saline solution and administered at eight points along the wound margin, while the control group received 0.2 mL of 0.9% saline solution at the same sites. Furthermore, wound tissues were collected for immunohistologic staining with antibodies against transforming growth factor-beta (TGF-β), vascular endothelial growth factor (VEGF), and CD31^+^on days 6 and 14. In the Melsmon-treated group, the reduction in wound size was accelerated from day 3 to day 9 compared with that in the control group. TGF-β and VEGF levels on days 6 and 14, respectively, were significantly increased in the Melsmon-treated group. Furthermore, CD31^+^ increased in the Melsmon-treated group as wound healing progressed. Based on these results, Hon et al. concluded that Melsmon promotes wound healing when directly administered to the wound edge.

Park et al. reported protective effects of Melsmon in rats exposed to benzo[*α*]pyrene (BaP) (Park et al., 2010). To investigate whether Melsmon protects lymphocytes from DNA damage caused by BaP, rats were divided among the following four groups: 1) control group (vehicle only), 2) Melsmon exposure group (20 μL × 3 times/week for 2 weeks, intramuscular injection), 3) BaP exposure group (200 mg/kg body weight, intraperitoneal injection) and 4) Melsmon + BaP group (20 μL × 3 times/week for 2 weeks, intramuscular injection followed by BaP at 200 mg/kg body weight, intraperitoneal injection). Lymphocytes were then isolated from rat whole blood and analyzed using the comet assay. In addition, the concentrations of superoxide dismutase (SOD), malondialdehyde (MDA), and carbonyls were measured to evaluate the antioxidant effects of Melsmon against BaP in rat plasma, and the levels of immunoglobulins and inflammatory cytokines, such as tumor necrosis factor-α (TNF- α), IL-1b, and IL-6, were measured to evaluate the anti-inflammatory effects of Melsmon treatment. In BaP exposure group, the olive tail moments determined by the comet assay were significantly higher than in the control group; however, Melsmon + BaP group, olive tail moments significantly decreased. Furthermore, pretreatment with Melsmon attenuates the BaP-induced elevation of SOD activity and reduces early-phase increases in MDA levels, indicating suppression of excessive enzymatic responses and lipid peroxidation. However, protein oxidation levels remain unchanged across all groups, suggesting that Melsmon’s protective effects do not extend to protein carbonylation. In addition, pretreatment with Melsmon significantly suppressed the levels of inflammatory cytokines, such as TNF-α, IL-1b, and IL-6. Based on these results, Park et al. concluded that pretreatment with Melsmon reduces BaP-induced DNA damage and may significantly inhibit the oxidative damage and inflammation caused by BaP (Park et al., 2010).

Oh et al. evaluated the effects of Melsmon treatment on mice exposed to gamma irradiation (Oh et al., 2023). They assessed DNA damage in lymphocytes and lymphoid organs, including lymph nodes, bone marrow, spleen, and thymus, of mice treated with Melsmon before and after gamma irradiation exposure, as well as of mice without Melsmon treatment. The results showed that, in the group of mice without Melsmon, gamma irradiation significantly increased the olive tail moments in the comet assays of blood lymphocytes and lymphatic organs and induced DNA damage, being the highest in lymphocytes and the lowest in the bone marrow. In contrast, when mice were administered Melsmon by intramuscular injection after gamma irradiation, DNA damage in the blood lymphocytes and lymph nodes was significantly suppressed. Furthermore, when Melsmon was administered by intramuscular injection before gamma irradiation, it significantly suppressed DNA damage in blood lymphocytes, lymph nodes, and bone marrow. Based on these results, Oh et al. concluded that Melsmon administration reduces DNA damage caused by gamma irradiation (Oh et al., 2023).

Additionally, Park et al. evaluated the therapeutic effects of Melsmon in two murine models of rheumatoid arthritis: KBx/N serum-transfer arthritis and collagen-induced arthritis (Park et al., 2012). Mice received intraperitoneal injections of Melsmon (1 µL or 100 μL, three times per week), and the incidence and severity of arthritis, hind-paw swelling, joint destruction, and histopathological alterations were assessed. Across both models, Melsmon did not result in significant improvements in clinical arthritis scores, paw thickness, radiologic findings, or histopathologic markers compared with vehicle controls. Moreover, levels of inflammatory cytokines including TNF-α, IL-1β, IL-6, IL-10, and RANKL, in serum and joint tissues showed no significant differences between groups (Park et al., 2012). These findings suggest that administration of Melsmon does not confer therapeutic benefits in the progression of experimental rheumatoid arthritis.

#### 4.1.3 Functions/effects confirmed by clinical studies

Clinical trial records using Melsmon registered in the ClinicalTrials.gov and WHO ICTRP databases were systematically retrieved and consolidated. Comprehensive details of these registrations are provided in Supplementary Table S1.

Kang et al. investigated the clinical efficacy of interfollicular injections of autologous platelet-rich plasma (PRP) containing CD34^+^ cells for pattern hair loss (Kang et al., 2014). PRP containing CD34^+^ cells or Melsmon was injected into the scalp of 13 patients (seven males and six females) with pattern hair loss, and the efficacy of these treatments was evaluated. Six months after the initial treatment, patients treated with PRP containing CD34^+^ cells showed clinical improvements with average increases in the number of hairs (29.2% ± 17.8%, P < 0.0001), hair thickness (46.4% ± 37.5%, P < 0.0001), and two-point scores (121.3% ± 66.8%, P < 0.0001). Besides, in those patients who received Melsmon-treatment, an improvement in the average number of hairs (26.0% ± 14.6%, P < 0.0001), hair thickness (21.4% ± 14.6%, P = 0.001), and two-point scores (72.8% ± 15.0%, P < 0.0001) was also observed when compared to the baseline (Kang et al., 2014).

Kim et al. conducted clinical trials evaluating the efficacy and safety of a pharmaceutical product on female subjects with menopausal symptoms at four facilities in South Korea (Kim et al., 2013; Kim et al., 2023). The administration regimen was as follows: one ampoule (2 mL) was administered per session, three times a week for a total of six doses, with injections given into subcutaneous tissues, such as the abdomen, upper arm, or thigh. Melsmon, which has been confirmed to be safe and effective not only in Japan but also in South Korea, was used as the control medicine in these trials. Either the investigational medicine or Melsmon was administered six times each, and the change in the Kupperman index, a menopause-related index, was evaluated 12 days later (primary efficacy evaluation). In addition, they analyzed the changes in hormone levels and menopausal symptoms (secondary efficacy evaluation). The changes in the Kupperman index in the Melsmon and investigational medicine groups were almost the same, and both Melsmon and the investigational medicine were effective in improving menopausal symptoms. In addition, there were no statistically significant differences between the two groups in blood test results or vital signs.

Kovalenko et al. reported the results of a randomized, double-blind, placebo-controlled, prospective clinical trial using a parallel group of Melsmon (Kovalenko and Atalyan, 2016). They recruited 40 premenopausal women with symptoms of menopausal syndrome, menstrual disorders, or follicle-stimulating hormone (FSH) levels higher than 20 mIU/mL and randomly assigned them to two groups (n = 20 each). The groups were administered either 2 mL of Melsmon or saline solution subcutaneously every other day for 2 weeks and then twice a week for a total of 30 administrations over 4 months. After the treatment, in the Melsmon-treated group, the Modified Kupperman Menopausal Index, the time required to fall asleep, and the number of nighttime awakenings decreased significantly, and the duration of sleep increased, compared to the results in the control group. In addition, the majority of the women participating in this clinical trial experienced improvements in mood and depressive symptoms as well as in their skin condition as a result of the administration of Melsmon.

Pokul et al. reported a clinical trial aimed at investigating the effects of Melsmon administration in patients with gynecological cancer with post-ovariectomy syndrome (Pokul et al., 2017). In total, 131 patients of reproductive age with cervical cancer, stages I–III carcinomas of the uterine corpus and ovary, and total hysterectomy were divided into three clinical groups. Group 1 (n = 43) included patients with cervical, uterine body, and ovarian cancer after combined complex treatment, surgical treatment, and chemotherapy, who received Melsmon therapy to reduce post-ovariectomy syndrome symptoms. Group 2 (n = 37) comprised patients with stage II–III cervical cancer after combined complex treatment, who also received Melsmon therapy to treat post-ovariectomy syndrome. The comparison group (Group 3; n = 51) included patients who received combined complex treatment for cervical, uterine body, and ovarian cancer as well as traditional symptomatic vitamin and sedative therapy without Melsmon administration for the treatment of post-ovariectomy pathologic manifestations. The progress of cancer and the possibility of recurrence were monitored using a real-time ultrasound scanner, and prospective clinical psychological tests were performed. Given that Melsmon has been registered in the Russian Federation since 2011 as a reformulated drug for use in women during menopause, its subcutaneous administration was carried out in accordance with the recommendations of the corresponding Russian drug registry. Patients were administered 2-mL doses of Melsmon subcutaneously every other day (three times a week) for 2 weeks and twice a week for the next 12 weeks (the total duration of treatment was 14 weeks). The patients were monitored by ultrasound imaging of the abdominal and pelvic organs. The results showed that there was no modification in the prognosis of the underlying disease due to Melsmon administration and that Melsmon administration helped improve the psychological state of patients with gynecological cancer by overcoming stress (Pokul et al., 2017).

Orazov et al. examined the effects of Melsmon on the improvement of genitourinary syndrome, a problem in modern gynecology (Orazov et al., 2017a). In this clinical trial, comprehensive treatment using subcutaneous Melsmon injections as metabolic therapy was administered to 62 women aged up to 60 years with clinical symptoms of genitourinary syndrome. Results showed a significant effect in reducing depressive symptoms and decreasing the mental distress scale index. Further, it was confirmed that sexual function was significantly improved in the group that received Melsmon, furthermore, Melsmon administration has a high clinical effect that improves the quality of life (QOL) of postmenopausal women. In a previous report by Kovalenko et al. on the use of HPE in women with climacteric syndrome during the perimenopausal period, subcutaneous administration of Melsmon alleviated menopausal symptoms such as hot flashes, insomnia, depression, and irregular skin changes, and was also associated with partial recovery (32%) of menstrual function in some cases (Kovalenko and Atalyan, 2016).

Orazov et al. also reported a randomized, prospective, non-comparative study that aimed to evaluate the efficacy of combined therapy with metabolic correction using dydrogesterone and Melsmon in patients with luteal phase deficiency (Orazov et al., 2017b). Women of reproductive age (n = 35) were administered a basic treatment of 10 mg of dydrogesterone twice a day from days 14–28 of the menstrual cycle for three cycles, and Melsmon (as a metabolic corrector) was administered subcutaneously twice a week from the first day of the menstrual cycle for 4 weeks for three cycles. Only 23 patients (65.7%) had dominant follicles confirmed by ultrasonography before treatment; however, after combined therapy, dominant follicles were detected in 33 patients (94.4%). In addition, it was confirmed that continuing combined therapy increased the luteal diameter from 1.36 ± 0.32 mm to 2.16 ± 0.21 mm and significantly increased the levels of estrogen and progesterone corresponding to each stage of the menstrual cycle. It was concluded that combined treatment with metabolic correction using dydrogesterone and Melsmon is a useful treatment method for restoring the cyclic regulation of the ovarian–menstrual cycle.

### 4.2 The functions/effects of Laennec

#### 4.2.1 Functions/effects confirmed *in vitro*

Wu et al. reported the effect of Laennec on concanavalin A (Con A)-induced liver injury (Wu et al., 2008b). They established an *in vitro* model of liver injury in which primary-cultured female rat hepatocytes and lymphocytes were cocultured and Con A was used to induce cell damage. Wu et al. evaluated the protective effect of Laennec by measuring the amount of leakage of hepatocyte cytoplasmic enzymes in this model. The addition of Laennec reduced the amount of leakage of the hepatic cytoplasmic enzymes aspartate aminotransferase (AST) and lactate dehydrogenase (LDH) compared to that in the control group without Laennec. They further purified *cyclo*-*trans*-4-*L*-hydroxyprolyl-*L*-serine from Laennec and identified it as the responsible for the hepatoprotective effect of the extract and for the reduction in leakage of AST, LDH, and TNF-α from hepatocytes (Wu et al., 2008a). Moreover, pretreatment of hepatocytes with *cyclo*-*trans*-4-*L*-hydroxyprolyl-*L*-serine also inhibits DNA fragmentation. In addition, immunocytochemistry and RT-PCR showed that the caspase-3 expression at the protein and mRNA levels in the *cyclo*-*trans*-4-*L*-hydroxyprolyl-*L*-serine-treated group was lower than those in the untreated group, and that the expression of intercellular adhesion molecule-1 (ICAM-1) was also suppressed by this dipeptide. These results showed that the hepatoprotective effect of Laennec is due to presence of *cyclo*-*trans*-4-*L*-hydroxyprolyl-*L*-serine which suppresses apoptosis and ICAM-1 expression in hepatocytes.

Liu et al. evaluated the hepatoprotective effects of the *trans*-4-*L*-hydroxyprolyl-*L*-serine and *cyclo*-*trans*-4-*L*-hydroxyprolyl-*L*-serine dipeptides found in Laennec by measuring the leakage of liver cytosolic enzymes from primary cultured rat hepatocytes treated with carbon tetrachloride (CCl_4_) (Liu et al., 2000). The addition of either dipeptide decreased glutamic oxaloacetic transaminase (GOT) and LDH activities in the culture medium in a dose-dependent manner. In addition, GOT activity was almost equivalent to that of hepatocytes without CCl_4_ poisoning in the culture medium when the highest concentration of any of the peptides was added. Based on these results, it was concluded that the dipeptides *trans*-4-*L*-hydroxyprolyl-*L*-serine and *cyclo*-*trans*-4-*L*-hydroxyprolyl-*L*-serine exhibit potent antihepatotoxicity effects in rat hepatocytes.

Inoue et al. reported a compound in Laennec involved in DNA synthesis stimulation (Inoue et al., 2015). They found that hydrolysates prepared with hydrochloric acid effectively promoted DNA synthesis in primary rat hepatocytes compared to those prepared with proteases, and discovered that pyroglutamic acid was the responsible for such DNA synthesis promotion via MAPK signaling in primary rat hepatocytes.

Ishikawa et al. performed *in vitro* experiments using primary mouse macrophages to elucidate the molecular mechanisms underlying the effects of Laennec (Ishikawa et al., 2025). Their results revealed that Laennec promoted the polarization of macrophages toward the anti-inflammatory M2 phenotype and activated anti-inflammatory signaling pathways. These findings suggest that Laennec directly modulates macrophage function and contributes to the suppression of inflammatory and senescence-associated cellular processes *in vitro*.

Yamaguchi et al. reported the compounds with anti-tumor activity contained in Laennec (Yamaguchi et al., 2016). To identify such compounds, they used the anti-proliferative effect on HepG2 cells as an indicator. They showed that Laennec inhibits the proliferation of HepG2 cells in a dose-dependent manner and that it is the combination of aspartic acid (Asp) and glutamic acid (Glu), the two most abundant amino acids in Laennec, that inhibits the growth of HepG2 cells in a dose- and time-dependent manner. In addition, they concluded that the anti-tumor activity induced by Asp + Glu was mediated via the Akt pathway, which plays an important role in the cellular regulatory network. Since Laennec improves liver damage through liver regeneration, suppression of inflammatory reactions, and inhibition of hepatocyte apoptosis, and is effective for treating patients with non-alcoholic steatohepatitis (NASH) who do not respond to lifestyle interventions, Yamauchi et al. also studied the effects of Laennec on rat hepatic stellate cells (Yamauchi et al., 2020). Treating rat hepatic stellate cells with Laennec significantly suppressed the expression of *Acta2*, *Col1a1,* and *Tgfβ1* genes and inhibited Smad phosphorylation; the treatment also increased the expression of antioxidant genes, such as *Hmox1*, *Nqo1*, *Cat,* and *Sod1*, enhanced NRF2 activity, decreased the expression of *Nox4*, and attenuated the levels of intracellular reactive oxygen species.

Shin et al. investigated the cellular effects of Laennec on tendon-derived fibrocytes isolated from rat ligament tissue (Shin et al., 2019). Treatment with Laennec promoted fibrocyte proliferation and maintained cell viability and macrophage activity at levels comparable to those of the normal control group. Furthermore, Laennec enhanced the expression of extracellular matrix-related genes, including *Col1*, *Scx*, and *Tnmd*, suggesting activation of fibroblastic differentiation and tissue remodeling pathways. These findings indicate that Laennec facilitates ligament regeneration by stimulating fibrocyte function and extracellular matrix synthesis.

#### 4.2.2 Functions/effects confirmed *in vivo*

Nakayama et al. performed partial hepatectomy on normal and CCl_4_ cirrhotic rats, and investigated the effects of Laennec treatment by intravenous and subcutaneous administration on the regeneration of the remaining liver. The results showed that both intravenous and subcutaneous administration of Laennec increased the rate of liver regeneration in normal rats and rats with CCl_4_-induced liver cirrhosis following partial hepatectomy (Nakayama et al., 1989a). In addition, intravenous administration of Laennec to normal rats with partial hepatectomy suppressed the decrease in hepatic protein and reduced serum transaminases (such as GOT and glutamate-pyruvate transaminase [GPT]), and that administration of Laennec to CCl_4_-induced cirrhotic rats with partial hepatectomy suppressed the increase in serum GOT. Furthermore, histological examination of the regenerated liver confirmed that intravenous administration of Laennec improved the vacuolization and necrosis of hepatocytes in normal and CCl_4_ cirrhotic rats with partial hepatectomy. It was concluded that Laennec administration was effective in promoting the regeneration of the remaining liver after partial hepatectomy.

Nakayama et al. also investigated the effects of intravenous and subcutaneous injections of Laennec in rats with CCl_4_-induced acute and chronic liver damage (Nakayama et al., 1989b). The results showed that intravenous and subcutaneous administration of Laennec to rats with acute and chronic liver damage induced by CCl_4_ suppressed the increase in serum (caused by CCl_4_) of both GOT and GPT. Moreover, in rats with acute or chronic liver damage, both treatments suppressed the vacuolization, swelling, and necrosis caused by the loss of cytoplasm and nuclei of perivascular cells in the central vein that results from CCl_4_ administration. Therefore, intravenous and subcutaneous administration of Laennec was effective in improving liver damage.

Using a similar CCl_4_-induced methods for murine model of liver cirrhosis, Ishikawa et al. demonstrated that administration of Laennec significantly improved liver function, as evidenced by reduced serum ALT levels and improved hepatic architecture (Ishikawa et al., 2025). Transcriptomic and histological analyses further showed that Laennec decreased the number of senescent cells in the liver and enhanced anti-inflammatory M2 macrophage polarization. These *in vivo* results indicate that Laennec exerts hepatoprotective and regenerative effects through immunomodulatory and anti-senescence mechanisms in the CCl_4_-induced liver injury model.

Using a murine model of liver injury, Wu et al. conducted *in vivo* studies on the anti-liver injury effects of Laennec (Wu et al., 2008b). They induced liver injury by injecting Con A into the tail veins of mice previously treated intramuscularly with Laennec, and the extent of the liver damage was then assessed. It was shown that Laennec administration reduced the activity of cytoplasmic enzymes, such as alanine aminotransferase (ALT) and LDH, in mice sera and restored SOD activity and MDA levels in mice liver tissues. Furthermore, while the Con A administration group showed a typical DNA ladder, the Laennec administration group showed significantly suppressed DNA ladders. Besides, the effect of Laennec on the expression of the apoptosis-associated genes *bcl-2* and *bax* was also investigated. While Con A administration significantly decreased the *bcl-2*/*bax* ratio, both the Laennec-treated and the control groups showed similar ratios. Together, the *in vitro* and *in vivo* studies suggest that Laennec may exert its hepatoprotective effect by suppressing inflammatory reactions and apoptosis.

Liu research group studied *in vivo* the hepatoprotective effects of the dipeptide *cyclo*-*trans*-4-*L*-hydroxyprolyl-*L*-serine isolated from Laennec (Yang et al., 2009), aiming to confirm the effects observed in *in vivo* studies reported by their group (Liu et al., 2000; Wu et al., 2008a). They used mice with Con A-induced liver injury that were orally administered *cyclo*-*trans*-4-*L*-hydroxyprolyl-*L*-serine before and after Con A administration. After 8 h, the activity of cytoplasmic enzymes, such as ALT and LDH, was measured, and either the activity or concentration of key enzymes, including superoxide dismutase, dialdehyde malonic acid, myeloperoxidase, and nitric oxide, in liver homogenates were also evaluated. Additionally, histological changes in the livers were analyzed. The results showed that the administration of *cyclo*-*trans*-4-*L*-hydroxyprolyl-*L*-serine significantly suppressed the activity of cytoplasmic and liver homogenate enzymes, and significantly reduced the concentrations of ICAM-1 and TNF-α. In addition, the increase in DNA fragmentation and decrease in the *bcl-2*/*bax* mRNA ratio induced by Con A were significantly suppressed by *cyclo*-*trans*-4-*L*-hydroxyprolyl-*L*-serine. These results showed that *cyclo*-*trans*-4-*L*-hydroxyprolyl-*L*-serine prevented immune-mediated liver injury, probably by the immunomodulation of T cells and adhesion molecules together with its antioxidant and antiapoptotic effects (Yang et al., 2009).

To study the anti-liver injury effects of *tran*s-4-*L*-hydroxyprolyl-*L*-serine and *cyclo*-*trans*-4-*L*-hydroxyprolyl-*L*-serine, Liu et al. conducted *in vivo* studies in rats with chemically-induced liver injury (Liu et al., 2000). Intravenous and oral administration of *trans*-4-*L*-hydroxyprolyl-*L*-serine and *cyclo*-*trans*-4-*L*-hydroxyprolyl-*L*-serine suppressed bilirubin and hepatic cytosolic enzyme activities. Together, these results and those from *in vitro* studies indicate that *trans*-4-*L*-hydroxyprolyl-*L*-serine and *cyclo*-*trans*-4-*L*-hydroxyprolyl-*L*-serine are useful compounds with high potential for clinical applications.

Yamauchi et al. investigated the effects of Laennec on hepatic fibrosis using *db/db* mice, which show obesity and insulin resistance and are thought to reproduce the pathological background of NASH, as model system (Yamauchi et al., 2020). Mice were fed a normal diet until 8 weeks of age and then either continued on the control diet or were switched to a methionine choline deficient (MCD) diet for an additional 8 weeks (challenge period). During this period, mice received intramuscular injections of 0.4 mL Laennec or control saline twice weekly. Although feeding with the MCD diet led to hepatic atrophy with periportal fibrosis, the development of periportal fibrosis was markedly suppressed in mice treated with Laennec. Furthermore, the number of alpha-smooth muscle actin (αSMA)-positive activated hepatic stellate cells increased in mice fed a MCD diet, but this phenomenon was reversed by the administration of Laennec. It was concluded that the administration of Laennec would effectively improve hepatic fibrosis in NASH.

To investigate the effects of Laennec on hepatic iron deposition, Yamauchi et al. developed a murine model of NASH with hepatic iron deposition (Yamauchi et al., 2019a). After feeding the mice an MCD diet supplemented with 2% iron for 12 weeks, iron deposition was detected in the liver and confirmed to spread throughout this organ. In addition, F4/80-positive macrophages accumulated in the liver, and the expression of genes related to inflammation and oxidative stress in the liver increased. During the MCD diet period, mice received intramuscular injections of Laennec (0.1 mL, 3.6 mg/kg) or control saline twice weekly. After administering Laennec to these model mice, iron deposition in the liver was significantly reduced and the accumulation of F4/80-positive macrophages decreased by about half over time. From these results, it was concluded that the administration of Laennec may effectively improve liver damage caused by iron overload by suppressing inflammation, oxidative stress, and iron deposition and promoting iron excretion.

Tumor growth suppression by Laennec was investigated *in vivo* by Yamaguchi et al. using a rabbit VX2 liver tumor model (Yamaguchi et al., 2016). They prepared an emulsion of lipiodol and aspartic and glutamic acids, the two most abundant amino acids in Laennec, and injected it into the hepatic artery. The treatment suppressed the growth of tumor cells in rabbit VX2 livers in a dose-dependent manner. The tumor growth ratio in the group given a low-dose emulsion was −26.3 ± 31.7, whereas that in the high-dose group was −33.9 ± 13.0. Based on these results, Yamaguchi et al. concluded that the combination of aspartic and glutamic acids is useful for inducing tumor cell death.

Park et al. investigated the biological effects of Laennec on a stress-induced depression animal model (Park et al., 2018). Rats were subjected to repeated immobility stress (4 h/d, 7 days) after the intramuscular administration of Laennec (0.02 mL, 0.2 mL or 1 mL/rat, 30 min before the daily stress session in Laennec group, while control groups were given sterile saline), and their depressive-like behavior was evaluated using the elevated plus maze (EPM) and forced swim test (FST). The effects of Laennec on NADPH-diaphorase (NADPH-d) and glutathione peroxidase (GPx) were also evaluated by enzyme-linked immunosorbent assay (ELISA) and immunohistochemistry (IHC), respectively. Laennec-treated mice showed significantly reduced immobility times in the FST, compared to that in the control group, and tended to have reduced anxiety behavior in the EPM. Furthermore, Park et al. showed that Laennec administration increased the level of GPx in the hippocampus and decreased the expression of NADPH-d (a nitric oxide [NO] synthase) in the paraventricular nucleus. From these results, Park et al. suggested that Laennec may have anti-stress effects by regulating NO synthase and antioxidant activity in the brain and concluded that Laennec would be useful in the treatment of stress-related diseases such as chronic fatigue syndrome (CFS).

Yamauchi et al. investigated the effects of Laennec in mice with angiotensin II (Ang II)-induced cachexia (Yamauchi et al., 2019b). They prepared an Ang II-induced cachexia model by continuously injecting Ang II into mice; Then, Laennec was administered intramuscularly (3.6 mg/kg once a day for 7 days) to these mice to investigate its effects on body weight and composition as well as on cardiac hypertrophy, inflammation, and fibrosis. Compared to the parameters of the control group, Laennec administration maintained both fat mass and lean body mass in cachexia model mice and also suppressed weight loss. In addition, Laennec suppressed the expression of BNP and βMHC genes (which are related to the heart failure induced by continuous injection of Ang II) and that of collagen α1, TGF-β, and matrix metallopeptidase 2 (MMP2) genes in the heart. Laennec significantly suppressed infiltrating inflammatory cells and the expression of the inflammatory-related molecules TNF-α and ICAM-1 in the heart. Yamauchi et al. concluded that the administration of Laennec may be effective in the treatment of cachexia.

Shin et al. conducted a study to identify the *in vivo* effects of Laennec on tendon and ligament injury in an animal model (Shin et al., 2019). Sprague Dawley rats were divided into a negative control (normal) group and a ligament injury group, and the ligament injury group was further divided into saline, Laennec, polydeoxyribonucleotide, and 20% glucose administration groups. The ligaments were collected at 1 and 4 weeks after treatment and subjected to various examinations. Unlike those in the saline and glucose administration groups, the number of cells and the level of activated macrophages in the Laennec administration group were maintained at the same level as in the normal group, and the level of degenerative changes was low even after 4 weeks of Laennec administration. The results showed that Laennec administration suppressed the immune response of damaged ligaments, promoting their healing, and suggest that Laennec is a useful candidate medicine for the treatment of injured ligaments (Shin et al., 2019).

Kim et al. evaluated the antiviral effects of Laennec against the novel coronavirus SARS-CoV-2, responsible for the COVID-19 pandemic (Kim et al., 2021). Experimental ferrets were infected with SARS-CoV-2 and then Laennec was administered intravenously (4 mL on days 0, 2, 4, and 6 post infection), and the clinical symptoms and viral titers in their respiratory tracts were compared with those of the control group (phosphate buffer solution administration group, days 0–4 post infection) and the positive control group (remdesivir, a representative COVID-19 drug, administration group, 17.6 mg/kg on day 1 as a loading dose and 8.8 mg/kg daily on days 2–4 as maintenance doses). The results showed that Laennec administration minimized weight loss after infection with COVID-19, reduced viral replication in the nasal wash fluid and lungs of infected ferrets, and had the same effect as the administration of remdesivir. Furthermore, it was revealed that the expression of type I interferons, IFN-α and IFN-β, and type II interferon, IFN-γ, was significantly increased in COVID-19-infected ferrets treated with Laennec compared to that in the control group. These results suggest that Laennec reduces the viral load and clinical symptoms of SARS-CoV-2-infected ferrets to the same extent as remdesivir.

#### 4.2.3 Functions/effects confirmed by clinical studies

Clinical trial records using Laennec registered in the ClinicalTrials.gov and WHO ICTRP databases were systematically retrieved and consolidated. Comprehensive details of these registrations are provided in Supplementary Table S1.

Glazachev et al. reported an evaluation of the efficacy and safety of Laennec in the treatment of patients diagnosed with CFS (Glazachev et al., 2017). A total of 38 patients diagnosed with CFS were divided into two groups, the Laennec and control groups. The Laennec group received an intravenous drip infusion of 4 mL of Laennec in 250 mL of saline solution twice a week, and no medicines were administered to the control group. The effects of Laennec were evaluated using a questionnaire to determine the degree of chronic fatigue in patients with CFS and the subjective evaluation of QOL. The survey of the Laennec and control groups was conducted before, just after, and 5 weeks after the Laennec administration. In addition, the physical endurance of patients allocated to both groups was evaluated using a cardiopulmonary exercise test. Before treatment with Laennec, there were no differences between the two groups in terms of the degree of chronic fatigue, anxiety, depression, and QOL, and the degree of chronic fatigue was moderate in both groups. In addition, the results of the subjective evaluation of patients' QOL showed that the scores for the measures of general health, vitality, mental health, physical pain, and social functioning were low in both groups. However, while no changes were seen in the control group, the Laennec group showed a decrease in chronic fatigue just after and 5 weeks after Laennec treatment, the degree of chronic fatigue was confirmed to decrease to a low-level category of CFS. In addition, after receiving Laennec treatment, the QOL of patients in the Laennec group significantly improved, and this trend continued for 5 weeks after the end of treatment. In addition, patients in the Laennec group showed improvements in measures of physical pain, vitality, emotional state, and mental health, and the degree of these improvements reached values close to those in healthy states. Furthermore, patients in the Laennec group showed an increase in physical endurance. Based on these results, Glazachev et al. suggested that Laennec treatment should be considered an option for patients with CFS.

Kim et al. conducted a single-blind, randomized controlled trial to evaluate the effectiveness and safety of Laennec, administered into the subacromial space in patients with shoulder impingement syndrome (Kim et al., 2025). 50 patients were randomized to receive either Laennec or placebo, and all participants underwent three weekly ultrasound-guided subacromial injections of 4 mL. The Laennec-treated group showed significant improvements in pain visual analog scale score, shoulder pain and disability index, and euroQoL 5-dimension 5-level utility index during the 9-week follow-up period after the final injection, in contrast to the placebo group. Time-dependent improvements across all outcome measures were observed only in the Laennec group. No major adverse events or clinically relevant abnormalities were reported. These findings indicate that Laennec is effective and well tolerated, suggesting its potential as an alternative therapeutic option for shoulder impingement syndrome.

### 4.3 The functions/effects of Placentrex

#### 4.3.1 Functions/effects confirmed *in vitro* and *in vivo*

Sur et al. aimed to clarify the antiplatelet aggregation and anti-inflammatory activities of Placentrex (Sur et al., 2003). Platelet aggregation was induced *in vitro* in platelet-rich plasma (PRP) prepared from human blood by adding 1 mmol/L adenosine diphosphate (ADP). Different amounts of Placentrex were added to PRP 5 min before the addition of ADP, and the effect of Placentrex on antiplatelet aggregation was measured by light transmittance. Platelet poor plasma (PPP) prepared from PRP was used as a control, and the results of platelet aggregation were expressed as the percentage of aggregation after 5 min of ADP addition. Placentrex, at all doses, showed a significant antiplatelet aggregation effect. Furthermore, the anti-inflammatory effects of Placentrex were investigated *in vivo* using Wistar rats either with carrageenan-, serotonin-, or prostaglandin E1-induced edema (acute model) and cotton pellet-induced granuloma (subacute model). The results showed that Placentrex administration significantly suppressed the edema induced by the three methods. Especially in the serotonin-induced edema model, the edema suppression rate by Placentrex was better than that observed with diclofenac sodium, an anti-inflammatory medicine. In addition, Placentrex significantly reduced the weight of granuloma tissue due to subacute inflammation, although the reduction rate was better in the diclofenac sodium-administered group. It was concluded that Placentrex may be useful for inhibiting inflammation and platelet aggregation.

#### 4.3.2 Functions/effects confirmed by clinical studies

Clinical trial records using Placentrex registered in the ClinicalTrials.gov and WHO ICTRP databases were systematically retrieved and consolidated. Comprehensive details of these registrations are provided in Supplementary Table S1.

Kondaveeti et al. evaluated the therapeutic effects of Placentrex on oral mucositis in patients with oral cancer undergoing treatment with concurrent chemoradiotherapy (Kondaveeti et al., 2018). Patients with oral cancer undergoing concurrent chemoradiotherapy were given 2 mL of Placentrex intramuscularly once a day for 4 weeks for treating oral mucositis. The administration of Placentrex delayed the progression of mucositis, reduced treatment interruptions, alleviated pain, and improved dysphagia, with no side effects observed. In conclusion, Placentrex is a beneficial treatment option for the management of acute oral mucositis induced by concurrent chemoradiation in patients with oral cancer.

A similar study was conducted by Prasad et al. (2024). They evaluated the efficacy of Placentrex in treating radiation-induced oral mucositis in patients with head and neck malignancies. In the study group, a dose of 2 mL of Placentrex was administered as a deep intramuscular injection into the deltoid muscle once daily, starting from the 11th fraction of radiation therapy and continued until completion, including both treatment and non-treatment days. The control group received only supportive and symptomatic management, including betadine or other mouthwashes, analgesic ointments, and nonsteroidal anti-inflammatory drugs. The percentage of patients with grade 2 or higher oral mucositis at the end of treatment for head and neck malignancies was 40% in the Placentrex administration group and 81% in the control group. Based on these results, Prasad et al. concluded that the administration of Placentrex has a significant effect in reducing the severity of radiation-induced mucositis, reducing the interruption or delay of treatment for head and neck malignancies.

The incidence of oral submucosal fibrosis (OSMF), which is characterized by excessive collagen production in the oral submucosa, is high in the Indian subcontinent and Far East. In most cases, OSMF leads to progressive fibrosis of the oral mucosa and atrophic epithelial changes. Over a 10-week period, Shinde et al. clinically evaluated the efficacy of local injections of Placentrex on OSMF, in comparison with that of local injections of triamcinolone acetonide, which is widely used as a treatment for oral mucosa diseases (Shinde et al., 2019). The results showed that there was a significant improvement in the symptoms of OSMF, including improvements in burning sensation, difficulty in opening the mouth, pain, and cheek flexibility, in both the Placentrex and triamcinolone acetonide groups.

A similar study was conducted by Kisave et al. (2020), who evaluated the effects of Placentrex administered by submucosal injection over 8 weeks in patients with OSMF and confirmed that Placentrex reduced burning sensation, which is one of the major symptoms of the disease (Kisave et al., 2020).

Reddy et al. evaluated the clinical efficacy of intraoral submucosal injections of Placentrex in comparison with corticosteroids for the management of OSMF (Reddy et al., 2022). In this 1-year hospital-based study involving 30 patients, both treatment modalities were assessed based on improvements in interincisal distance and histopathological changes. Although the results were not statistically significant, patients treated with Placentrex exhibited slightly better improvement in mouth opening and fibrosis reduction compared with those receiving corticosteroids. Further, Ek et al. conducted a prospective clinical study to compare the therapeutic efficacy of intralesional Placentrex versus hyaluronidase combined with dexamethasone injections in patients with stage II OSMF (Ek et al., 2023). Over a 14-month follow-up, 15 patients were evaluated in each group for changes in mouth opening, burning sensation, and mucosal color. Both treatment modalities significantly improved symptoms, but the group receiving Placentrex showed better improvement in mouth opening and greater reduction in burning sensation than the hyaluronidase + dexamethasone group (*p* < 0.001). These findings indicated that intralesional Placentrex is a safe and effective option for reducing fibrosis and improving oral function in OSMF patients.

## 5 Discussion

As mentioned in this review, in addition to improving menopausal symptoms, promoting lactation, and improving liver function, various placental extracts, including human placental extracts, are used for anti-aging purposes, such as the treatment of skin wrinkles (Yoshikawa et al., 2013). However, their mechanisms of action are not yet fully understood. For this reason, many studies have carried out to clarify the anti-aging effects of placental extracts. For example, research on nicotinamide adenine dinucleotide (NAD), which is known to decrease with age, suggests that maintaining NAD levels can prevent and treat aging and age-related diseases (Rajman et al., 2018; Yoshino et al., 2018), and studies have been conducted to identify the factors that increase intracellular NAD levels in placental extracts (Katayoshi et al., 2022). In addition, recent studies have shown that chronic inflammation associated with aging, known as inflammaging, may accelerate the aging process (Franceschi et al., 2000; Le Saux et al., 2012; Fulop et al., 2023), and it is hypothesized that suppressing chronic inflammation can lead to anti-aging effects. As shown in this review, various placental extracts have anti-inflammatory effects. Therefore, further research on the anti-inflammatory effects of placental extracts is warranted to provide science-based anti-aging medicines.

Recent improvements in analytical technology have made it possible to analyze trace components in samples. Lately, Torshin et al. determined the sequences of 41 peptides contained in Laennec (Torshin et al., 2023). In addition, Shen et al. showed that 128 peptides derived from 85 proteins were present in placental extracts prepared from bovine placentas hydrolyzed with papain (Shen et al., 2024). To date, some of biological functions of placental extracts containing various peptides have been reported. For example, dipeptides such as Gly-Leu and Leu-Gly found in porcine placental extracts (PPE) have been reported to promote the expression of the brain-derived neurotrophic factor (BDNF) gene in Caco-2 cells used as an intestinal epithelial cell model. BDNF is suggested to play an important role in mental stress (Nagae et al., 2024). The BDNF produced in the gut is thought to activate neuronal cells, thereby forming an intestine-to-brain regulatory pathway of neural function. Enhanced production of BDNF and nerve growth factor (NGF) has been associated with the promotion of neuronal growth and maintenance, as well as with improvements in various brain functions (Allen et al., 2011), including improvements in sleep disturbances. Furthermore, Han et al. investigated the anti-fatigue effects of PPE–derived dipeptides, including Gly-Leu and Leu-Gly, using treadmill exercise tests and forced swimming tests in mice (Han et al., 2018). Oral administration of these dipeptides significantly prolonged time to exhaustion, enhanced BDNF levels and phosphorylated extracellular signal-regulated kinase signaling in the brain, and increased muscle and liver glycogen content. Gly-Leu and Leu-Gly also reduced markers of exercise-induced fatigue, such as lactate dehydrogenase, lactate, creatine kinase, blood urea nitrogen, alanine transaminase, and aspartate transaminase, in serum and muscle. Inflammatory responses were attenuated through suppression of proinflammatory cytokine production, inhibition of caspase-1 activation, and reduction of nuclear factor-κB signaling in activated splenocytes. These findings indicate that PPE-derived dipeptides mitigate physical fatigue by enhancing dopaminergic activity, increasing energy metabolism, and suppressing.

On the other hand, as mentioned in this review, it was reported that administration of Placentrex, one of the human placental extracts, alleviated pain and improved dysphagia in a clinical study (Kondaveeti et al., 2018). Based on the pain relief and improvement of dysphagia observed with the Placentrex administration, it is strongly presumed that Transient receptor potential (TRP) channels, a type of ion channel, would be affected by Placentrex. TRP channels, which are located in nerve endings, are involved in pain and burning sensations (Caterina et al., 2000; Davis et al., 2000) as well as in swallowing (Ebihara et al., 2006; Hossain and Kitagawa, 2023). Therefore, placental extracts may contain agonist- or antagonist-like components that activate TRP channels.

Previously, we have reported that peptide quorum sensing molecules (Tobita et al., 2022) involved in communication among in same kind of bacteria (Kitaguchi and Swartz, 2005; Andreev et al., 2013; Monastyrnaya et al., 2016; Tobita et al., 2022) modulated TRPV1 and TRPA1 functions. On the contrary, it was reported that cow placental extracts, which contain various active peptides with immunomodulatory and antioxidant effects, were administered orally to immunosuppressed mice with cyclophosphamide-induced intestinal damage, the treatment improved intestinal microbiota composition, intestinal damage, and immune function (Zhao et al., 2024). The improvement in the intestinal microbiota might be the result of the peptides contained in the cow placental extract functioning as quorum-sensing molecules for intestinal bacteria. These results suggest that placental extracts containing diverse peptides hold promising potential for regulating fundamentally different crucial biological phenomena, such as TRP channel modulation and improvement of the gut microbiota.

## 6 Perspectives

Although generally considered safe, HPE preparations can cause certain adverse reactions that warrant medical attention, as documented in the official product information. The most frequently reported events are local injection site reactions, including pain, redness, swelling, and induration, which are typically mild and transient; however, repeated administration at the same site may occasionally result in persistent nodules or sclerosis. Systemic adverse reactions are rare and usually reflect hypersensitivity responses, manifesting as rash, pruritus, fever, or malaise. The overall incidence of such events remains low, although isolated reports of severe allergic reactions, including anaphylaxis, have been described. Additionally, transient hepatic enzyme elevation or hormone related symptoms, such as breast swelling, have occasionally been observed, though their causal relationships remain uncertain. Since these preparations are derived from human placenta, a theoretical risk of infection transmission exists despite rigorous donor screening, viral inactivation, and sterilization processes during manufacturing; nevertheless, no such cases have been reported to date. Taken together, HPE preparations appear to possess a favorable safety profile when used under appropriate medical supervision. Continuous pharmacovigilance and mechanistic studies, however, remain essential to better understand rare adverse events and ensure long-term safety.

The chemical composition contained in each HPE varies depending on the manufacturing method, but they contribute to cellular proliferation, tissue regeneration, and antioxidant defense mechanisms. Despite their long-standing clinical application, the complete chemical composition of these extracts remains incompletely characterized, and many of their active components and molecular mechanisms of action are still unidentified. Comprehensive chemical and functional analyses are therefore crucial to elucidate the pharmacological basis of these preparations and establish a scientific foundation for their efficacy and safety. By correlating the putative bioactive molecules predicted from the biological effects observed in experimental and clinical studies with the identified chemical entities such as peptides and low molecular weight compounds, it may become possible to determine the principal active ingredients responsible for their therapeutic effects. Such investigations are expected to reveal novel biological functions and expand the potential clinical applications of placental extracts. Ultimately, these studies will contribute to the standardization of placental formulations and promote their evidence-based integration into the fields of regenerative medicine and anti-aging research.
